# Melatonin inhibits Sirt1-dependent NAMPT and NFAT5 signaling in chondrocytes to attenuate osteoarthritis

**DOI:** 10.18632/oncotarget.18356

**Published:** 2017-06-03

**Authors:** Jia Yi Guo, Feng Li, Yong Bing Wen, Hong Xun Cui, Ma Long Guo, Lin Zhang, Yun Fei Zhang, Yan Jin Guo, Yan Xing Guo

**Affiliations:** ^1^ Luoyang Orthopedic Hospital of Henan Province, Luoyang, Henan, China; ^2^ Department of Surgery, Advanced Clinical Skills Centre, University of Auckland, Auckland, New Zealand

**Keywords:** melatonin, redox, Sirt1, NAMPT, NFAT5, Immunology and Microbiology Section, Immune response, Immunity

## Abstract

Osteoarthritis (OA) is a degenerative joint disease mainly characterized by cartilage degradation. Interleukin-1β (IL-1β) contributes to OA pathogenesis by enhancing oxidative stress and inflammation. Melatonin reportedly elicits potent protection against OA. However, the role of melatonin and underlying mechanism in IL-1β-stimulated chondrocytes remain largely unclear. In this study, we found that melatonin inhibited IL-1β-induced toxicity and sirtuin 1 (Sirt1) enhancement in human chondrocytes. Melatonin reduced the IL-1β-increased nicotinamide phosphoribosyltransferase (NAMPT) expression and the NAD^+^ level in chondrocytes in a Sirt1-dependent manner. In turn, the inhibitory effect of melatonin on Sirt1 was mediated by NAMPT. Moreover, melatonin suppressed IL-1β-induced Sirt1-mediated matrix metalloproteinase (MMP)-3 and MMP-13 production. Melatonin also decreased the Sirt1-steered nuclear factor of activated T cells 5 (NFAT5) expression in IL-1β-challenged chondrocytes. NFAT5 depletion mimicked the suppressive effects of melatonin on IL-1β-elevated production of inflammatory mediators, including tumor necrosis factor-α (TNF-α), IL-1β, prostaglandin E2 (PGE_2_), and nitric oxide (NO) in chondrocytes. TNF-α, IL-1β, PGE_2_, or NO decrease caused the similar reduction of MMP-3 and MMP-13 by melatonin in IL-1β-insulted chondrocytes. Highly consistent with in vitro findings, in vivo results demonstrated that melatonin repressed the expression of relevant genes in rat OA pathogenesis in anterior cruciate ligament transection model. Overall, these results indicate that melatonin effectively reduced IL-1β-induced MMP production by inhibiting Sirt1-dependent NAMPT and NFAT5 signaling in chondrocytes, suggesting melatonin as a potential therapeutic alternative for chondroprotection of OA patients.

## INTRODUCTION

Osteoarthritis (OA) is one of the most common joint diseases mainly characterized by synovium inflammation and cartilage degradation [1]. OA is highly prevalent among the elderly population and is also one of the three most common causes of disability in younger people [2]. Although OA therapy has significantly improved, the optimal treatment has yet to be developed [3]. Thus, identifying a novel therapeutic strategy of OA is urgently needed.

OA is generally accepted to be caused by deregulation of catabolic and anabolic processes that regulate cartilage matrix synthesis, despite that the etiology of OA has not been completely elucidated. Chondrocytes, the only cell type present in cartilage, are responsible for producing and maintaining the extracellular matrix (ECM) [4]. Accumulating evidence suggests that inflammatory mediators produced by chondrocytes play critical roles in the development of OA [5]. Interleukin-1β (IL-1β) and tumor necrosis factor-α (TNF-α) are the two most potent catabolic factors that increase the production of inflammatory mediators and the expression of matrix metalloproteinases (MMPs) in chondrocytes [6, 7]. MMPs greatly contribute to ECM degradation in OA [8]. Therefore, inhibiting IL-1β production and IL-1β-induced inflammation in chondrocytes may be useful for OA therapy.

Mounting studies have demonstrated the important roles of sirtuin 1 (Sirt1), a nicotinamide adenine dinucleotide (NAD^+^)-dependent histone deacetylase, in the pathogenesis of OA [9, 10]. Terauchi et al. [10] reported that Sirt1 is ubiquitously observed in osteoarthritic chondrocytes and regulates the development of chondrocyte hypertrophic lineage and progression of cartilage degeneration upon Runx2-mediated MMP-13 production in OA. In the literature, Sirt1 induces nicotinamide phosphoribosyltransferase (NAMPT) expression [11]. NAMPT is the rate-limiting enzyme that catalyzes the first step of NAD^+^ biosynthesis from nicotinamide [12] and NAMPT expression is increased in inflamed synovial tissues of mice as well as in the plasma and synovial fluids of OA patients [13]. Meanwhile, NAMPT can enhance the expression of MMPs in chondrocytes to promote cartilage erosion [14]. Intriguingly, NAMPT enzymatic activity stimulates the synthesis of NAD^+^, which is an essential cofactor of Sirt1 deacetylases [15]. Thus, disrupting Sirt1-NAMPT-NAD^+^-Sirt1 positive feedback loop signaling may prevent OA progression.

Sirt1 transcriptionally activates the expression of nuclear factor of activated T cells 5 (NFAT5) [16]. NFAT5, known as tonicity-responsive enhancer binding protein, is originally identified as a tonicity-regulated transcription factor involved in the protection of cells from hypertonic stress [17]. Other than hypertonicity, pro-inflammatory cytokines, including TNF-α and IL-1β, stimulated both expression and nuclear localization of NFAT5 in the fibroblast-like synoviocytes from OA patients [18]. NFAT5 can induce the expression of key pro-inflammatory factors, such as TNF-α, IL-1β, IL-6, cyclooxygenase-2 (COX-2), inducible nitric oxide synthase (iNOS), and MMP-13, in articular cartilage tissues [19]. Studies showed that MMP-3 expression is dependent on TNF-α and IL-1β in the nucleus pulposus cells affected with inflammatory disc disease [20]. Prostaglandin E2 (PGE_2_) and nitric oxide (NO) can also induce MMP expression in chondrocytes to degrade cartilage [21, 22]. Thus, inhibition of Sirt1-dependent NFAT5 signaling may lead to chondroprotection against OA.

Melatonin (*N*-acetyl-5-methoxytryptamine) is a hormone produced in all vertebrates including the humans [23, 24], which exhibits diverse biological activities, such as anti-oxidation, anti-inflammation, and anti-apoptosis [25, 26]. Melatonin elicits protective effects in different models of OA [27–29]. Melatonin can reduce the inhibitory effect of TNF-α and IL-1β on the chondrogenesis of mesenchymal stem cells; this phenomenon is potentially associated with the ability of melatonin to scavenge free radicals, preserve superoxide dismutase, and suppress MMPs generation [28]. Reportedly, melatonin treatment combined with treadmill exercise prevents collagenase-induced cartilage damage by exerting anti-inflammatory and anti-apoptotic effects [29]. Melatonin also enhances matrix synthesis in IL-1β-challenged porcine articular chondrocytes [27]. Lim et al. [9] demonstrated that melatonin possesses protective effects on human chondrocytes by the Sirt1 pathway involved in inflammation and oxidative stress. However, the suppressive effects of melatonin and underlying mechanism in catabolic responses of OA chondrocytes to oxidation and inflammation have not been fully identified.

In this study, we investigated whether melatonin elicits protective effects in IL-1β-stimulated chondrocytes. The findings show that melatonin reduces IL-1β- induced oxidation- and inflammation-mediated MMP production in chondrocytes by inhibiting Sitr1-regulated NAMPT and NFAT5 signaling, suggesting that melatonin may be a novel effective therapeutic agent for chondroprotection of OA patients.

## RESULTS

### Melatonin inhibits IL-1β-induced toxicity and the expression and activity of Sirt1 in chondrocytes

**Figure 1:**
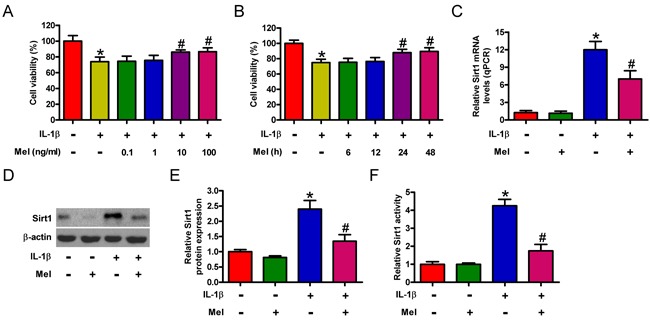
Melatonin reduced the expression and activity of Sirt1 in IL-1β-stimulated chondrocytes **A.** and **B.** Human chondrocytes were subjected to (A) treatment with varied concentrations of melatonin (0, 0.1, 1, 10, and 100 ng/ml) for 24 h or (B) treatment with 10 ng/ml melatonin for different durations (0, 6, 12, 24, and 48 h). **C.**-**F.** Chondrocytes were treated with or without 10 ng/ml IL-1β for 30 min, followed by treatment with 10 ng/ml of melatonin for 24 h. C and D. mRNA and protein levels of Sirt1 were measured by qPCR (C) and Western blot (D) assays, respectively. E. Quantification of Sirt1 protein expression in (D). GAPDH and β-actin were used as internal controls. F. Sirt1 activity was determined. Each value represents mean ± SD of 3 replicates or representative of 3 independent experiments. *P < 0.05 compared with control; ^#^*P* < 0.05 compared with IL-1β-treated group. Mel: melatonin.

We evaluated the protective effect of melatonin on IL-1β-exposed human chondrocytes. IL-1β significantly reduced the chondrocyte viability, whereas melatonin (0.1, 1, 10, and 100 ng/ml) alleviated IL-1β-induced cytotoxicity in a dose-dependent manner. 10 and 100 ng/ml melatonin markedly restored the viability of IL-1β-insulted chondrocytes; however, there were no marked differences in 10 and 100 ng/ml groups (Figure 1A). Moreover, 10 ng/ml melatonin (6, 12, 24, and 48 h) improved the viability of IL-1β-challenged chondrocytes in a time-dependent manner. The viability of chondrocytes increased after 24 and 48 h of melatonin treatment; however, no significant differences existed in 24 and 48 h groups (Figure 1B). Thus, treatment with 10 ng/ml melatonin for 24 h was selected for subsequent experiments. To investigate whether Sirt1 involves the response of IL-1β-stimulated chondrocytes to melatonin treatment, we examined the mRNA and protein expression as well as the activity of Sirt1. As shown in Figures 1C-1E, the IL-1β-induced mRNA and protein expression of Sirt1 was decreased by approximately 12- and 2.5-folds by melatonin, respectively. Melatonin also suppressed the Sirt1 activity by about fourfold in IL-1β-treated chondrocytes compared with that in control group (Figure 1F). Hence, melatonin inhibited IL-1β-induced expression and activity of Sirt1 in chondrocytes.

### Melatonin reduces NAMPT and NAD+ levels in a Sirt1-dependent manner in IL-1β-stimulated chondrocytes

**Figure 2:**
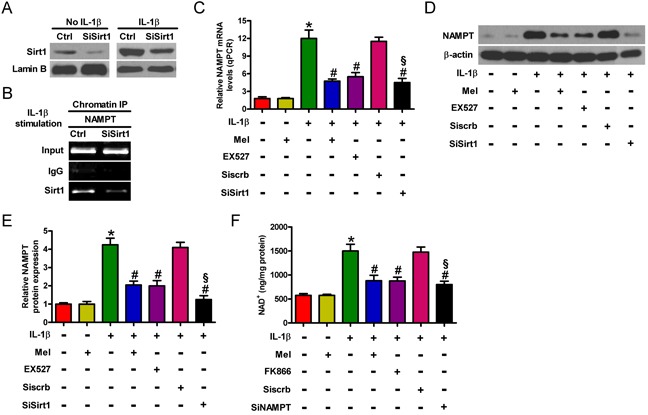
Melatonin inhibited IL-1β-activated Sirt1-NAMPT/NAD+ signaling in chondrocytes **A.** Mock- or IL-1β-treated chondrocytes were pretreated with 100 nM siSirt1 and incubated in serum-free media overnight. Sirt1 protein expression was measured by Western blot. Lamin B was used as internal control. **B.** IL-1β-stimulated chondrocytes were pretreated with 100 nM siSirt1 and incubated in serum-free media overnight. ChIP assay was performed. **C.**-**E.** Chondrocytes were pretreated with 100 μM Sirt1 inhibitor (EX527) for 30 min or 100 nM siSirt1 for 1 h and then stimulated with or without 10 ng/ml IL-1β for 30 min, followed by 10 ng/ml melatonin for 24 h. qPCR (C) and Western blot (D and E) analyses were performed to determine the mRNA and protein expression of NAMPT, respectively. GAPDH and β-actin were used as internal controls. F. NAD^+^ level in chondrocytes treated with 100 μM NAMPT inhibitor (FK866) for 30 min or 100 nM siNAMPT for 1 h and then stimulated with or without 10 ng/ml IL-1β for 30 min, followed by 10 ng/ml melatonin for 24 h. Each value represents mean ± SD of 3 replicates or representative of 3 independent experiments. *P < 0.05 compared with control; ^#^*P* < 0.05 compared with IL-1β-treated group; ^§^*P* < 0.05 compared with Siscrb group. Mel: melatonin.

NAMPT is transcriptionally upregulated by Sirt1 in the hepatocytes of obese mice [11]. We here investigated the role of Sirt1 in regulating the NAMPT gene in chondrocytes. As shown in Figure 2A, Sirt1 expression was significantly inhibited by siSirt1 treatment in chondrocytes with or without IL-1β insult. And the results of chromatin immunoprecipitation (ChIP) assay indicated that the levels of Sirt1 at the NAMPT gene promoter were markedly decreased by downregulation of Sirt1. Accordingly, expression of NAMPT gene was reduced in Sirt1-silenced IL-1β-challenged chondrocytes (Figure 2B). Moreover, we probed the effect of melatonin on NAMPT expression in IL-1β-stimulated chondrocytes. qPCR and Western blot analyses showed about 12- and 4-fold increases in the mRNA (Figure 2C) and protein (Figures 2D and 2E) expression of NAMPT in IL-1β-treated group compared with that in the control group. Melatonin significantly suppressed the upregulation of NAMPT at the transcriptional and translational levels (Figures 2C-2E). We next explored whether the reduction of NAMPT by melatonin is reliant on Sirt1 in IL-1β-challenged chondrocytes. Pretreatment with the Sirt1 inhibitor (EX527) or siSirt1 successfully repressed NAMPT mRNA and protein expression (Figures 2C-2E). IL-1β significantly increased the NAD^+^ level (from 520 ng/mg protein to 1480 ng/mg protein; Figure 2F), which was markedly abated by melatonin or the NAMPT inhibitor (FK866) or siNAMPT. Notably, we demonstrated that melatonin impeded IL-1β-enhanced expression and activity of Sirt1 in chondrocytes (Figures 1C-1F). Hence, melatonin decreased the levels of NAMPT and NAD^+^ by regulating Sirt1 in IL-1β-stimulated chondrocytes.

### Inhibitory effect of melatonin on Sirt1 is partially dependent on NAMPT in IL-1β-insulted chondrocytes

**Figure 3:**
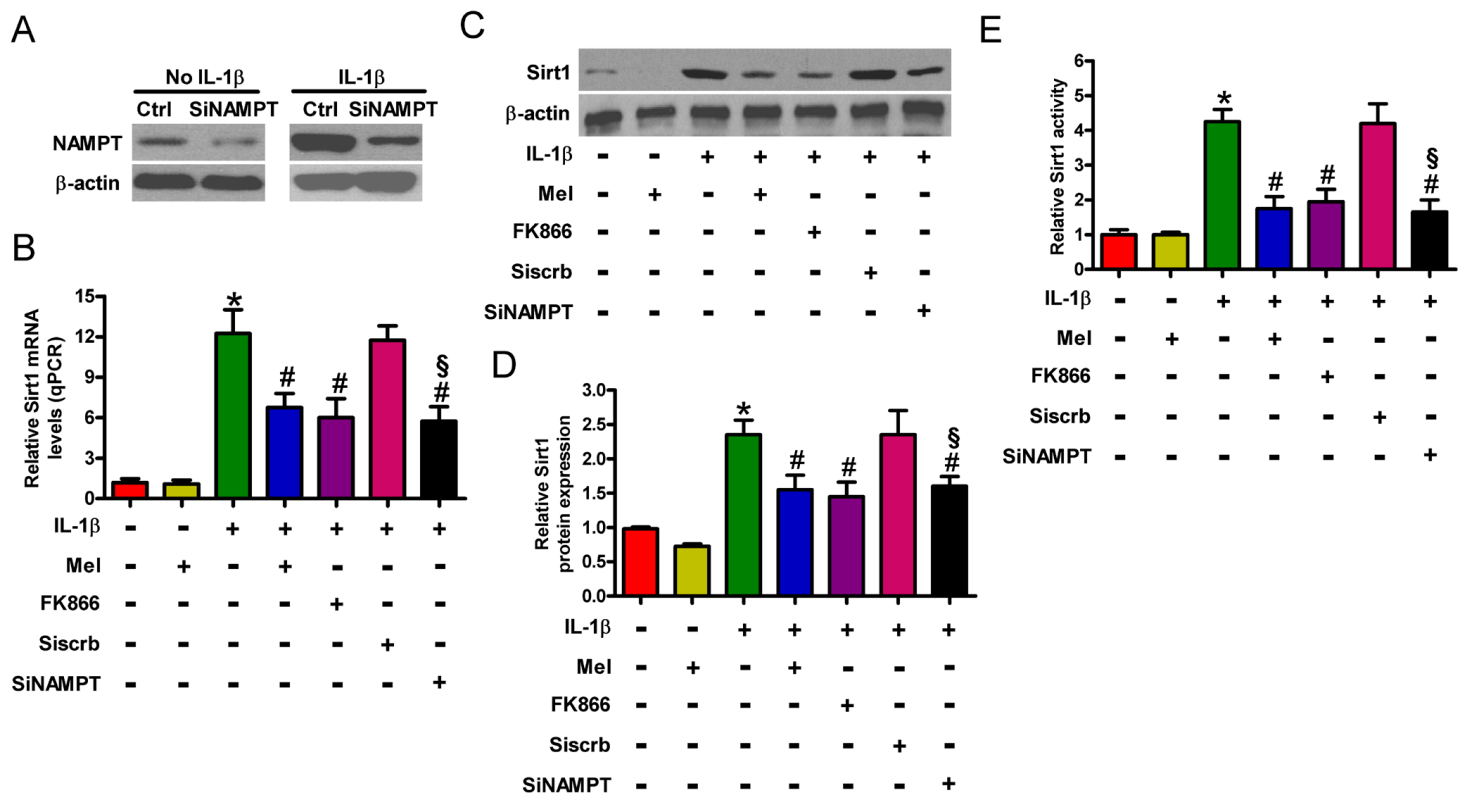
Melatonin decreased NAMPT-dependent Sirt1 expression in IL-1β-insulted chondrocytes **A.** Mock- or IL-1β-treated chondrocytes were pretreated with 100 nM siNAMPT and incubated in serum-free media overnight. NAMPT protein expression was measured by Western blot. β-actin was used as internal control. **B.**-**E.** Chondrocytes were pretreated with 100 μM NAMPT inhibitor (FK866) for 30 min or 100 nM siNAMPT for 1 h and then stimulated with or without 10 ng/ml IL-1β for 30 min, followed by 10 ng/ml melatonin for 24 h. (B) mRNA and (C) protein levels of Sirt1 were measured by qPCR and Western blot assays, respectively. GAPDH and β-actin were used as internal controls. D. Quantification of Sirt1 protein expression in (C). E. Sirt1 activity was determined. Each value represents means ± SD of 3 replicates or representative of 3 independent experiments. *P < 0.05 compared with control; ^#^*P* < 0.05 compared with IL-1β-treated group; ^§^*P* < 0.05 compared with Siscrb group. Mel: melatonin.

A previous study showed that NAMPT and Sirt1 form a positive regulatory loop that controls the NAD^+^ level [11]. Sirt1 is a downstream molecule of the NAMPT-mediated NAD^+^ biosynthesis pathway in chondrocytes [30]. To evaluate whether Sirt1 is involved in the protective effect of melatonin on IL-1β-stimulated chondrocytes in an NAMPT-dependent manner, we assessed the mRNA and protein expression as well as the activity of Sirt1 in the presence of the NAMPT inhibitor (FK866) or siNAMPT. As shown in Figure 3A, NAMPT expression was markedly reduced by siNAMPT treatment in mock- or IL-1β-treated chondrocytes. The mRNA and protein levels of Sirt1 decreased under FK866 or siNAMPT pretreatment in IL-1β-stimulated chondrocytes (Figures 3B-3D). FK866 or siNAMPT pretreatment also significantly reduced the increase in the activity of Sirt1 in IL-1β-stimulated chondrocytes (Figure 3E). Notably, we demonstrated that melatonin impeded IL-1β-enhanced expression and activity of NAMPT in chondrocytes (Figures 2C-2F). Thus, inhibition of Sirt1 expression and activity by melatonin was partly dependent on NAMPT in IL-1β-exposed chondrocytes.

### Melatonin suppresses MMP-3 and MMP-13 production by inhibiting Sirt1 expression and activity in IL-1β-challenged chondrocytes

**Figure 4:**
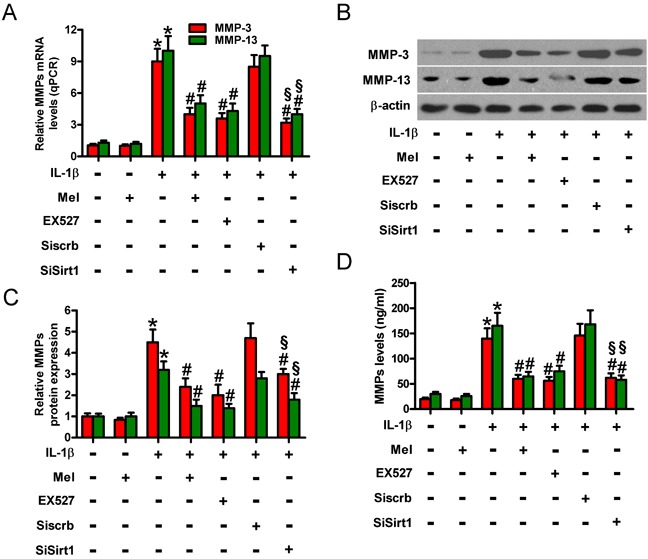
Melatonin reduced Sirt1-mediated MMP-3 and MMP-13 production in IL-1β-treated chondrocytes Chondrocytes were pretreated with 100 μM Sirt1 inhibitor (EX527) for 30 min or 100 nM siSirt1 for 1 h and then stimulated with or without 10 ng/ml IL-1β for 30 min, followed by 10 ng/ml melatonin for 24 h. **A.** qPCR and **B.** and **C.** Western blot analyses were performed to determine the mRNA and protein expression of MMP-3 and MMP-13, respectively. GAPDH and β-actin were used as internal controls. **D.** MMP-3 and MMP-13 levels in the media were measured by ELISAs. Each value represents mean ± SD of 3 replicates or representative of 3 independent experiments. *P < 0.05 compared with control; ^#^*P* < 0.05 compared with IL-1β-treated group; ^§^*P* < 0.05 compared with Siscrb group. Mel: melatonin.

MMP-3 and MMP-13 were upregulated in articular chondrocytes of OA patients [30]. As shown in Figure 4A, melatonin decreased IL-1β-induced mRNA expression of MMP-3 and MMP-13 by approximately 9- and 10-fold, respectively. Consistent with these results, the Western blot analysis revealed that melatonin reduced the enhancement of MMP-3 and MMP-13 protein (Figures 4B and 4C). In addition, melatonin significantly inhibited the IL-1β-induced production and release of MMP-3 and MMP-13 (Figure 4D). Reportedly, the inhibition of Sirt1 by nicotinamide or the Sirt1 inhibitor (EX527) caused to downregulation of MMP-3 and MMP-13 [30]. To address whether melatonin suppresses the expression of MMP-3 and MMP-13 by modulating Sirt1 in chondrocytes, we performed a series of experiments. As shown in Figures 4A-4D, EX527 or siSirt1 led to decrease in the mRNA and protein levels as well as the release of MMP-3 and MMP-13 in IL-1β-stimulated chondrocytes. Intriguingly, we found that melatonin hindered IL-1β-increased expression and activity of Sirt1 in chondrocytes (Figures 1C-1F). Therefore, melatonin reduced the production of MMP-3 and MMP-13 by inhibiting Sirt1 in IL-1β-treated chondrocytes.

### Sirt1 is involved in the inhibition of NFAT5 expression by melatonin in IL-1β-exposed chondrocytes

**Figure 5:**
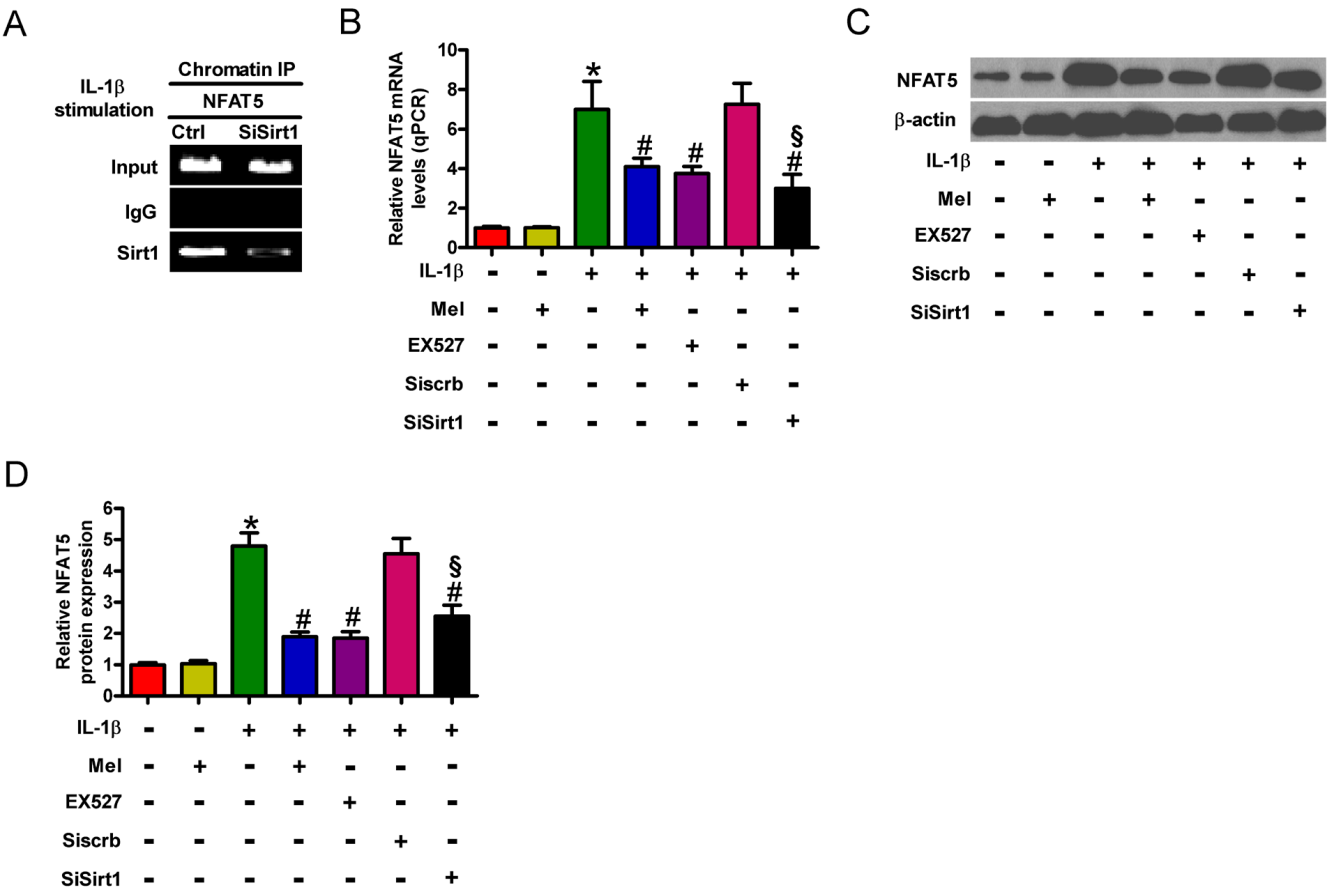
Melatonin inhibited Sirt1-upregulated NFAT5 expression in IL-1β-challenged chondrocytes **A.** IL-1β-exposed chondrocytes were pretreated with or without 100 nM siSirt1 and incubated in serum-free media overnight, and ChIP assay was performed. **B.**-**D.** Chondrocytes were pretreated with 100 μM Sirt1 inhibitor (EX527) for 30 min or 100 nM siSirt1 for 1 h and then stimulated with or without 10 ng/ml IL-1β for 30 min, followed by 10 ng/ml melatonin for 24 h. (B) qPCR and (C and D) Western blot analyses were performed to determine the mRNA and protein expression of NFAT5, respectively. GAPDH and β-actin were used as internal controls. Each value represents mean ± SD of 3 replicates or representative of 3 independent experiments. *P < 0.05 compared with control; ^#^*P* < 0.05 compared with IL-1β-treated group; ^§^*P* < 0.05 compared with Siscrb group. Mel: melatonin.

NFAT5 is highly expressed in OA chondrocytes [18] and is positively regulated by Sirt1 [16]. ChIP assay was performed to assess the role of Sirt1 in the regulation of NFAT5 in IL-1β-insulted chondrocytes. As shown in Figure 5A, levels of Sirt1 at the NFAT5 gene promoter were markedly reduced by siSirt1 treatment. We investigated the effect of melatonin on NFAT5 expression in IL-1β-stimulated chondrocytes. The qPCR assay showed that NFAT5 mRNA expression was enhanced by approximately 7-fold in IL-1β-treated chondrocytes compared with that in the control, which was markedly attenuated by melatonin (Figure 5B). Western blot analysis revealed that melatonin also inhibited IL-1β-increased NFAT5 protein expression (Figures 5C and 5D). We next investigated whether the reduction of NFAT5 by melatonin is dependent on Sirt1 in IL-1β-challenged chondrocytes. As shown in Figures 5B-5D, EX527 or siSirt1 pretreatment significantly reduced IL-1β-enhanced mRNA and protein levels of NFAT5. These results indicate that Sirt1 participated in the inhibitory effects of melatonin on IL-1β-induced NFAT5 expression in chondrocytes.

### Anti-inflammatory effects of melatonin is mediated by NFAT5 in IL-1β-treated chondrocytes

**Figure 6:**
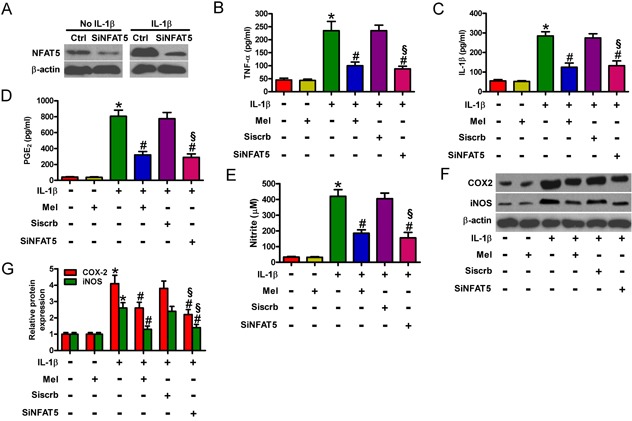
Melatonin elicited the suppressive effects on NFAT5-amplified inflammation in IL-1β-exposed chondrocytes **A.** Mock- or IL-1β-treated chondrocytes were pretreated with 100 nM siNFAT5 and incubated in serum-free media overnight. NFAT5 protein expression was measured by Western blot. β-actin was used as internal control. **B.**-**G.** Chondrocytes were pretreated with 100 nM siNFAT5 for 1 h and then stimulated with or without 10 ng/ml IL-1β for 30 min, followed by incubation with 10 ng/ml melatonin for 24 h. Levels of (B) TNF-α, (C) IL-1β, and (D) PGE_2_ in the media were measured by ELISAs. E. NO production was assessed using Griess reagent. F and G. Representative Western blot results (F) and quantification of COX-2 and iNOS expression (G). β-actin was used as internal control. Each value represents means ± SD of 3 replicates or representative of 3 independent experiments. *P < 0.05 compared with control; ^#^*P* < 0.05 compared with IL-1β-treated group; ^§^*P* < 0.05 compared with Siscrb group. Mel: melatonin.

NFAT5 promoted the expression of several inflammatory factors, such as TNF-α, COX-2, and iNOS [19]. We used siNFAT5 to determine the role of NFAT5 in the anti-inflammatory effects of melatonin on IL-1β-insulted chondrocytes. NFAT5 expression was remarkably decreased by siNFAT5 in IL-1β-exposed chondrocytes (Figure 6A). As predicted, siNFAT5 pretreatment significantly reduced the production of TNF-α (Figure 6B), IL-1β (Figure 6C), PGE_2_ (Figure 6D), and NO (Figure 6E), as well as the expression of COX-2 and iNOS (Figures 6F and 6G). To investigate the anti-inflammatory effects of melatonin, we assessed the production of TNF-α, IL-1β, PGE_2_, and NO as well as the protein expression of COX-2 and iNOS in IL-1β-treated chondrocytes. Compared with those in the control group, the levels of TNF-α, IL-1β, PGE_2_, and NO, as well as the expression of COX-2 and iNOS were increased significantly in the IL-1β-exposed group (Figures 6B-6G). However, melatonin markedly inhibited IL-1β-induced TNF-α, IL-1β, PGE_2_, and NO production (Figures 6B-6E). The protein expression of COX-2 and iNOS was also reduced by melatonin treatment (Figures 6F and 6G). Interestingly, we revealed the inhibition of IL-1β-induced NFAT5 expression by melatonin in chondrocytes (Figures 5B-5D). These results suggest that NFAT5 was involved in the anti-inflammatory effects of melatonin on IL-1β-stimulated chondrocytes.

### Melatonin-reduced MMP-3 and MMP-13 is reliant on TNF-α, IL-1β, PGE2, or NO decrease in IL-1β-challenged chondrocytes

**Figure 7:**
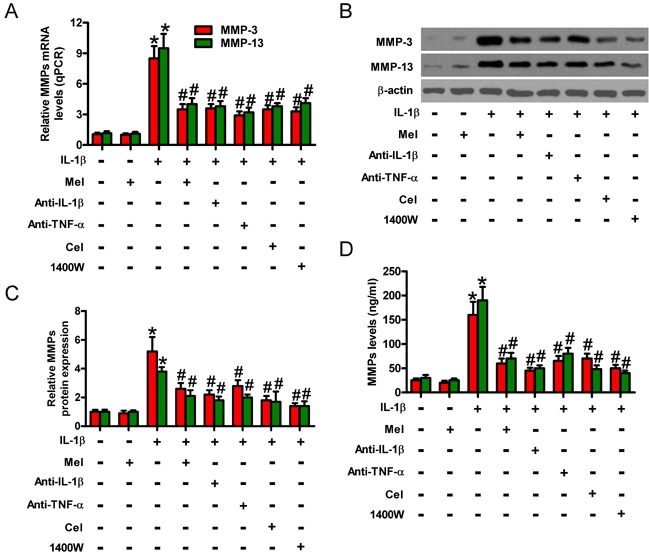
Melatonin-decreased MMP-3 and MMP-13 production was mediated by TNF-α, IL-1β, PGE2, and NO in IL-1β-stimulated chondrocytes Chondrocytes were pretreated with anti-TNF-α antibody (50 μg/ml), anti-IL-1β antibody (50 μg/ml), celecoxib (5 μM), or 1400W (50 μM) for 1 h and then stimulated with or without 10 ng/ml IL-1β for 30 min, followed by 10 ng/ml melatonin for 24 h. **A.** qPCR and **B.** and **C.** Western blot analyses were performed to determine the mRNA and protein expression of MMP-3 and MMP-13, respectively. GAPDH and β-actin were used as internal controls. **D.** Levels of MMP-3 and MMP-13 in the media were measured by ELISAs. Each value represents means ± SD of 3 replicates or representative of 3 independent experiments. *P < 0.05 compared with control; ^#^*P* < 0.05 compared with IL-1β-treated group. Mel: melatonin; Cel: celecoxib.

MMPs are elevated in chondrocytes of OA patients and greatly contribute to OA pathogenesis [6, 7]. Figure 7A shows that IL-1β induced significant increase in the mRNA expression of MMP-3 and MMP-13, which was attenuated by melatonin treatment. IL-1β-increased protein expression (Figures 7B and 7C) and the production and release (Figure 7D) of MMP-3 and MMP-13 in chondrocytes were also reduced by melatonin. Previous studies showed that TNF-α, IL-1β, PGE_2_, or NO may promote MMP production [20–22]. To determine whether the inhibition of MMP-3 and MMP-13 by melatonin is mediated by TNF-α, IL-1β, PGE_2_, or NO in IL-1β-stimulated chondrocytes, we used antibodies of TNF-α or IL-1β and inhibitors of COX-2 (celecoxib) or iNOS (1400W). Anti-TNF-α body, anti-IL-1β body, celecoxib, or 1400W pretreatment significantly reduced IL-1β-induced enhancement of MMP-3 and MMP-13 in chondrocytes (Figures 7A-7D). These results indicate that the reduction of MMP-3 and MMP-13 by melatonin was partly mimicked by neutralization of TNF-α and IL-1β or decrease of PGE_2_ and NO in IL-1β-stimulated chondrocytes.

### Melatonin ameliorates rat OA in anterior cruciate ligament transection model

**Figure 8:**
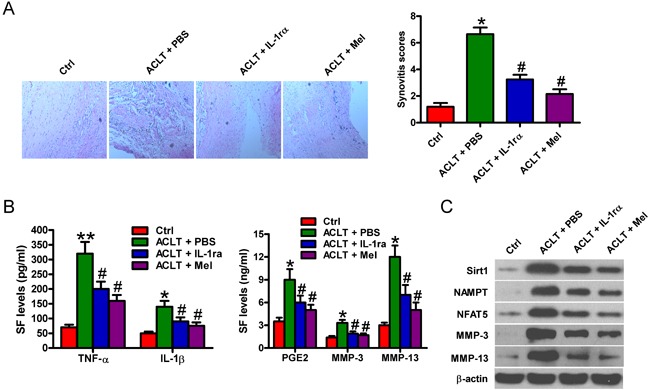
Melatonin impedes rat OA in ACLT model ACLT was performed in male Lewis rats. Animals were treated with PBS or IL-1 receptor antagonist (IL-1 ra) or melatonin (n = 6 in each group). **A.** HE analysis and staining evaluation was performed for synovitis scoring in PBS- or melatonin- or IL-1 ra-treated groups. (100 × magnification) **B.** ELISA assays were conducted to determine the SF levels of TNF-α, IL-1β, PGE_2_, MMP-3, and MMP-13 in three groups. **C.** Western blot analyses were performed to determine the protein expression of Sirt1, NFAT5, NAMPT, MMP-3 and MMP-13 in ACLT rat chondrocyte with or without melatonin or IL-1 ra treatment. β-actin was used as internal control. Each value represents means ± SD of 3 replicates or representative of 3 independent experiments. *P < 0.05, **P < 0.01 compared with control group; ^#^*P* < 0.05 compared with ACLT + PBS group. ACLT: anterior cruciate ligament transaction; Mel: melatonin; IL-1 ra: IL-1 receptor antagonist.

Next, we investigated whether melatonin retards synovial inflammation and cartilage degeneration in pre-clinical surgically-induced OA models. Anterior cruciate ligament transection (ACLT) was performed in male Lewis rats. Animals were treated with PBS or IL-1 receptor antagonist (IL-1 ra) or melatonin (n = 6 in each group). Histological analyses showed that, like IL-1 ra treatment, melatonin significantly reduced synovial scores compared to PBS (Figure 8A). As shown in Figure 8B, melatonin treatment reduced the SF levels of TNF-α, IL-1β, PGE_2_, MMP-3, and MMP-13 in the melatonin-treated group compared to the phosphate-buffered saline (PBS)-treated group, which was similar with IL-1 ra-treated group. Additionally, melatonin and IL-1 ra treatments have similar suppressive effects on the expression of several inflammation- or catabolic-related molecules in ACLT rat chondrocytes, such as Sirt1, NFAT5, NAMPT, MMP-3 and MMP-13 (Figure 8C). These results indicated that melatonin treatment ameliorated rat OA following ACLT.

## DISCUSSION

**Figure 9:**
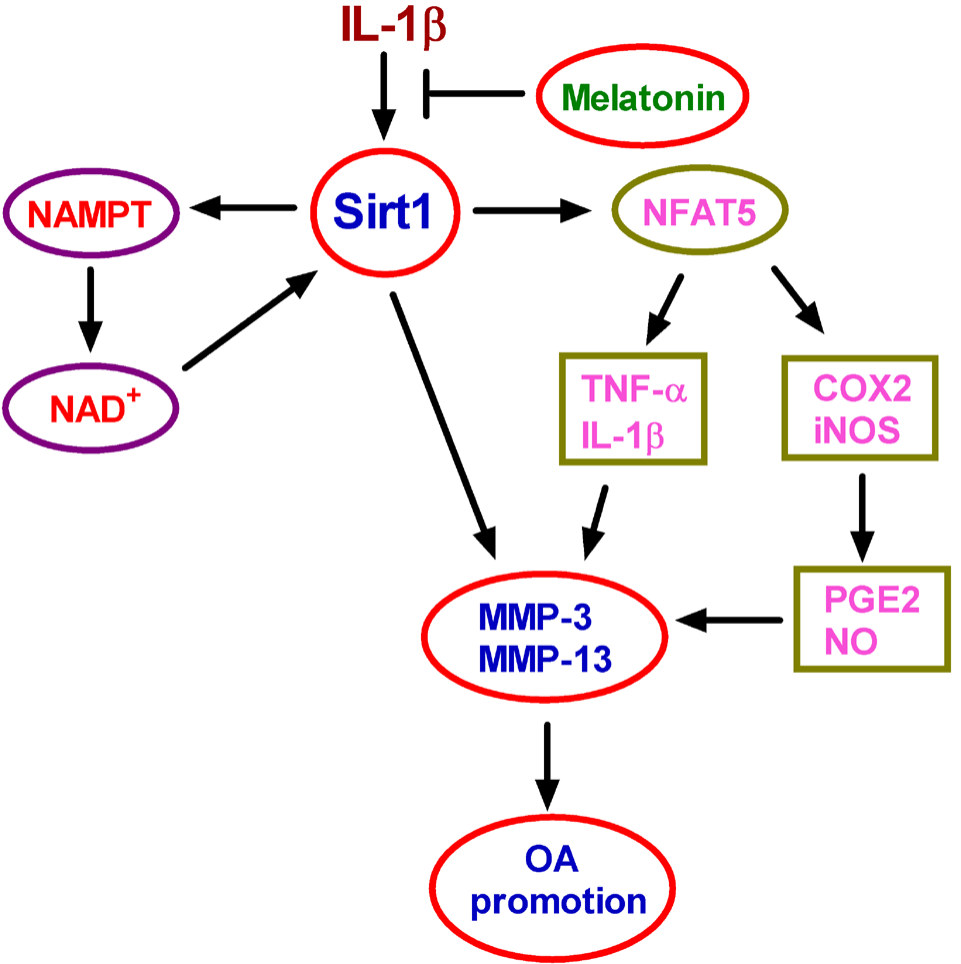
Schematic diagram of the proposed signaling pathways Melatonin reduced IL-1β-induced MMP-3 and MMP-13 production through Sirt1-dependent NAMPT and NFAT5 signaling in chondrocytes, suggesting melatonin as an efficient therapeutic agent to hinder OA progression.

In this study, we investigated the inhibitory effects of melatonin on IL-1β-induced MMP production in chondrocytes and attempted to elucidate the molecular mechanisms underlying such effects. The findings are presented as follows (Figure 9): (1) Treatments with melatonin of various concentrations or for different durations were performed to test cell viability; the results revealed the pro-survival effect of melatonin on IL-1β-stimulated chondrocytes in a dose- and time-dependent manner. (2) Melatonin inhibited the IL-1β-increased expression and the activity of Sirt1 in chondrocytes. (3) Melatonin antagonized the IL-1β-induced increase in the NAMPT/NAD^+^ levels. (4) Melatonin exhibited an NAMPT-dependent inhibitory effect on Sirt1. (5) Melatonin suppressed IL-1β-induced MMP-3 and MMP-13 production by regulating Sirt1/NAMPT/NAD^+^ signaling. (6) Melatonin reduced the IL-1β-induced Sirt1-mediated upregulation of NFAT5. (7) Melatonin inhibited IL-1β-induced NFAT5-mediated production of inflammatory mediators. (8) Melatonin-reduced MMP-3 and MMP-13 production was dependent on TNF-α, IL-1β, PGE_2_, and NO decrease in IL-1β-stimulated chondrocytes. (9) Melatonin show anti-inflammatory and anti-catabolic properties by regulating the expression of relevant genes in OA pathogenesis and progression, counteracting pro-inflammatory signals that lead to synovial inflammation and cartilage destruction. Overall, these findings corroborate that melatonin may benefit IL-1β-challenged chondrocytes in terms of anti-inflammatory and anti-catabolic effects, and the Sirt1-dependent NAMPT and NFAT5 signaling pathway may be involved in the actions of melatonin in chondrocytes.

Sirt1, as NAD^+^-consuming enzyme, is required in several important signal transduction pathways in mammalian cells [31]. Sirt1 plays an important role in the pathogenesis of OA. Although many literatures demonstrated Sirt1 exerts protective effects on OA, there are still some conflicting opinions concerning the association between Sirt1 and OA progression. Sirt1 provides a positive function in human cartilage by elevating expression of genes encoding cartilage extracellular matrix [32, 33]. Takayama et al. [34] reported that Sirt1 is downregulated in OA patients and regulates human chondrocytes apoptosis by modulating mitochondria-related apoptotic signals. Sirt1 may also promote the chondrogenic differentiation of mesenchymal stem cells [35]. In addition, Matsuzaki et al. [36] showed that cartilage-specific deletion of Sirt1 accelerated aging and injury-induced OA in mice. In contrast to these findings, a recent study suggested that Sirt1 is ubiquitously observed in osteoarthritic chondrocytes of OA and promotes MMP-13 production by regulating Runx2 expression [10]. Inhibition of Sirt1 with nicotinamide or EX527 blocked the upregulation of matrix-degrading enzymes (MMP-3, MMP-9, MMP-12, and MMP-13) in primary cultured chondrocytes induced by HIF-2α or NAMPT, suggesting that Sirt1 activity is necessary for the OA cartilage destruction [30]. Consistent with these observations, we here found that the expression and activity of Sirt1 increased in IL-1β-stimulated chondrocytes, and inhibition of Sirt1 attenuated the expression and production of MMP-3 and MMP-13. And the inconsistency with other previous findings may be resulted from the variable donors, the osteophyte formation, and the different treatments of the cells, etc. Further studies are needed to clarify the contradiction.

NAD^+^ (oxidative status)/NADH (reductive status) ratio is a key player in mediating mitochondrial energy metabolism by donating electrons to the electron transport chain or by acting as a coenzyme for rate-limiting enzymes of the tricarboxylic acid cycle [37]. NAMPT, as the rate-limiting enzyme in NAD^+^ biosynthesis, is serially upregulated in IL-1β-treated chondrocytes [38]. In this study, NAMPT expression and NAD^+^ level were enhanced by IL-1β challenge in chondrocytes, but were reduced by melatonin treatment. A previous study demonstrated that Sirt1 can induce NAMPT expression by binding to its promoter in the hepatocytes of obese mice [11]. Interestingly, Sirt1 activation is dependent on NAMPT in IL-1β-exposed articular chondrocytes [38], suggesting that Sirt1 and NAMPT may form a positive feedback system to activate downstream signaling. Consistent with these findings, our data showed that pretreatment of chondrocytes with the Sirt1 inhibitor (EX527) or siSirt1 significantly reduced the NAMPT expression and NAD^+^ level. Moreover, pretreatment with the NAMPT inhibitor (FK866) or siNAMPT inhibited the expression and activity of Sirt1 in chondrocytes. These results indicate that Sirt1 and NAMPT may form a positive regulatory loop in which Sirt1 induces NAMPT expression, leading to increased NAD^+^ levels and enhanced Sirt1 expression and activity. In a previous study, EX527 treatment blocked NAMPT-mediated upregulation of MMPs (MMP-3, MMP-9, MMP-12, and MMP-13) in primary cultured chondrocytes [30]. Reportedly, pretreatment with EX527 or siSirt1 reduced the production of MMP-3 and MMP-13 in IL-1β-challenged chondrocytes. These results confirm that melatonin disrupts Sirt1-dependent NAMPT signaling and suppresses MMP-3 and MMP-13 expression and production by scavenging NAD^+^-dependent Sirt1. The disruption was mediated by NAMPT in IL-1β-challenged chondrocytes, suggesting that melatonin inhibited MMP production by negatively regulating the Sirt1-NAMPT-NAD^+^-Sirt1 positive loop signaling to hinder OA progression.

NFAT5 is highly expressed in OA tissues and contributes greatly to the pathogenesis of OA [18]. NFAT5 is positively regulated by Sirt1 [16]. In this study, NFAT5 was considerably upregulated by IL-1β stimulation in chondrocytes, but this increase was reduced by melatonin. Inhibition of Sirt1 by its inhibitor (EX527) or siSirt1 reduced IL-1β-induced NFAT5 upregulation, suggesting that melatonin inhibits Sirt1-dependent NFAT5 expression in IL-1β-stimulated chondrocytes. Esensten et al. [39] demonstrated that NFAT5 induced the expression of TNF-α in a hypertonic culture by binding its promoter via the TonE element. The upregulation of TNF-α, IL-6, COX-2, and iNOS in macrophages is required for NFAT5 [40]. NFAT5 influences a number of targets relevant to matrix homeostasis, such as MMP-13 [40]. NFAT5 is also associated with the inflammation and ECM degeneration in OA pathogenesis [19]. In this study, NFAT5 depletion reduced the production and release of TNF-α, IL-1β, PGE_2_, and NO as well as the expression of COX-2 and iNOS. Inflammatory cytokines, such as IL-1β and TNF-α, were elevated in patients with OA [41]. IL-1β and TNF-α are strongly associated with the disruption of ECM components [42]. IL-1β can induce the production of TNF-α, PGE_2_, and NO, amplifying the inflammation and leading to the progression of OA [43]. Stimulating chondrocytes with IL-1β could upregulate the expression of COX-2 and iNOS, thereby promoting PGE_2_ and NO production [44]. PGE_2_ is implicated in the degeneration of articular cartilage [45, 46]. NO acts as an important reactive nitrogen species and plays a critical role in the pathogenesis of OA, leading to intensive oxidative stress and ultimately inducing chondrocyte and synoviocyte death [47]. NO can also induce the production of PGE_2_ and other pro-inflammatory cytokines [48]. Previous findings stated that melatonin exerts cytoprotective effects on OA progression by inhibiting inflammatory response and oxidative stress [9, 28]; consistently, we demonstrated that melatonin inhibited IL-1β-induced increase in TNF-α, IL-1β, PGE_2_ and NO production as well as COX-2 and iNOS expression in chondrocytes. Moreover, TNF-α, IL-1β, PGE_2_, and NO can increase the production of MMPs, which could degrade the components of the ECM [20–22]. In this study, we found that neutralization of TNF-α and IL-1β by using their corresponding antibodies or reduction of PGE_2_ and NO with the inhibitors of COX-2 (celecoxib) and iNOS (1400W) significantly reduced MMP-3 and MMP-13 production in IL-1β-challenged chondrocytes. Hence, melatonin exerted anti-inflammatory and anti-catabolic effects on IL-1β-stimulated chondrocytes. These results indicate that melatonin reduces the levels of inflammatory mediators such as TNF-α, IL-1β, PGE_2_, and NO in IL-1β-insulted chondrocytes by blocking Sirt1-dependent NFAT5 signaling, ultimately inhibiting inflammation-caused MMP-3 and MMP-13 production.

The current study has several limitations. (1) We should select more time points to investigate the more detailed molecular events *in vitro* and *in vivo*. (2) The study should be extended to investigate whether there is statistical difference between control groups and groups with IL-1β + melatonin (or IL-1β + other inhibitors) and whether melatonin, etc. block all or partial IL-1β response. (3) Reportedly, IL-1β induced Sirt1 decrease and inactivation in human OA chondrocytes [49]. By contrast, we found that IL-1β stimulation enhanced Sirt1 expression and activity in human chondrocytes, possibly attributing to individual variability. Our statistical evidence may be insufficient because the present study encompassed a relatively small number of OA patients. Thus, the enrollment of a larger number of patients will be beneficial to corroborate the involvement of Sirt1 in OA pathogenesis. (4) The OA disease process is complex to the extent that a single treatment may not confer the optimal chondroprotective effect to reduce cartilage degeneration. The use of combined treatment modalities may exert a synergistic effect leading to a better control of the OA disease progression. Nevertheless, we didn't investigate whether there was any additive effect of treating with melatonin along with other reagents. (5) Our results show that melatonin taps on important markers in OA and indicate that OA cartilage might benefit from the supplementation of melatonin. The *in vivo* part of the study could be extended to further confirm the *in vitro* findings, for example, by *in vivo* application of Sirt1 inhibitor, NAMPT inhibitor or COX2 inhibitor and monitoring of some pro-inflammatory parameters and cartilage degeneration. (6) The *in vitro* findings might not completely translate to an *in vivo* situation. Limitations including aging, types of animal, treatment protocol, and the timing periods of administration are critical for evaluating the therapeutic effects of melatonin. Thus, more investigations should be performed to grant melatonin as a potential therapeutic target of OA. (7) Many studies confirm the implication of several pro-inflammatory mediators, such as IL-1β and TNF-α, in the progression of OA. Blocking the effects of these key pro-inflammatory cytokines may prove useful in retarding the onset of cartilage degeneration and eventual OA. However, we here didn't not address whether melatonin had any inhibitory effect on TNF-α-mediated catabolism. Further studies are needed to confirm melatonin involvement in the effect of TNF-α.

In summary, melatonin reduces IL-1β-enhanced MMP production in chondrocytes by disrupting Sitr1-dependent NAMPT and NFAT5 signaling and simultaneously inhibiting the production of pro-inflammatory factors, suggesting that melatonin elicits anti-inflammatory and anti-catabolic activities and may be a potential therapeutic agent for OA.

## MATERIALS AND METHODS

### Ethics statement

All experimental procedures were approved by the Institutional Review Board of Luoyang Orthopedic Hospital of Henan Province and Henan Provincial Orthopedic Hospital. Written informed consent was obtained for all patient samples. Animal experiments were approved by the Institutional Committee for Animal Research and were performed in conformity with national guidelines for the care and use of laboratory animals.

### Reagents and antibodies

Melatonin, recombinant human IL-1β, trypsin, collagenase II, FK866, EX527, celecoxib, 1400W, 3-(4,5-dimethylthiazol-2-y1)-2,5-diphenyltetrazolium bromide (MTT), and dimethylsulfoxide (DMSO) were purchased from Sigma-Aldrich (St. Louis, MO, USA). Dulbecco's modified Eagle medium (DMEM), fetal bovine serum (FBS), 2-(4-(2-hydroxyethyl)-1-piperazinyl) ethanesulfonic acid (HEPES), penicillin, and streptomycin were obtained from Gibco BRL (Grand Island, NY, USA). Culture flasks and other disposable plastic were acquired from BD Bioscience (San Jose, CA, USA). Enzyme-linked immunosorbent assay (ELISA) kits for human or rat TNF-α, IL-1β, PGE_2_, MMP-3, and MMP-13 were supplied by R&D systems (Minneapolis, MN, USA). Mouse anti-human monoclonal antibodies (COX-2, iNOS, TNF-α, and IL-1β) were provided by Abcam (Cambridge, MA, USA). Rabbit anti-human or -rat polyclonal antibodies (Sirt1, NFAT5, NAMPT, MMP-3, and MMP-13) were obtained from Santa Cruz Biotechnology (Dallas, TX, USA). Mouse anti-human or -rat monoclonal antibody β-actin and mouse anti-human Lamin B were procured from Sigma-Aldrich. Horseradish peroxidase-conjugated anti-mouse or anti-rabbit IgG was purchased from Chemicon (Temecula, CA, USA). All other reagents were acquired from Sigma-Aldrich unless otherwise stated.

### Establishment of ACLT rat OA model

Transection of the ACL was performed in 9-10 weeks old male Lewis rats (n = 18). Following anesthesia with intraperitoneal Ketamine and Dexmedetomidine, the right knee joint skin was shaved, cleaned with povidone iodine topical antiseptic, and a lateral skin incision was made along the patellar tendon to access the joint capsule and expose the ACL. Severing the ACL was performed using a #11 surgical blade and a positive anterior draw confirmed ACLT. Closure of the joint capsule and animal skin was performed using biodegradable PDSII sutures. In all animals, the right knee joint was the ACL transected joint. All surgeries were performed by KE. Control animals (n = 6) were age and sex-matched to the ACL transected animals and were used for comparison.

### Intra-articular administration of melatonin, IL-1 ra, or PBS

To evaluate the impact of melatonin or IL-1 ra treatment on OA following ACLT, ACL transected animals were injected with 20 μl of melatonin (10 mg/ml) or 40 μl of recombinant human IL-1 ra (supplied as 150 mg/ml Anakinra, Amgen Inc, Thousand Oaks, CA) or PBS on day 3 following ACLT (n = 6 in each group). Injections were performed through the patellar tendon of the operated knee joint while the animal is receiving inhalational isoflurane. At 3 weeks post ACLT, lavaging of synovial fluid (SF) was performed by injecting a total of 100 μl of PBS into the joint capsule followed by flexing and extending the joint for 10 times. We successfully obtained lavages in 6 animals that had ACLT + PBS, in 6 animals that had ACLT + melatonin, in 6 animals that had ACLT + IL-1 ra and 6 control animals for ELISA assays. Then, animals were sacrificed and their joints were washed using PBS followed by joint harvest. The harvested joints were used for histological analyses and chondrocyte isolation as described below.

### Histological analyses and staining evaluation

Paraffin-embedded sections were taken from weight-bearing areas of the articular cartilage of ACL transected joints of each animal. Hematoxylin and eosin (H&E) staining was carried out for synovitis scoring. Synovial histopathology scoring was performed using the criteria reported previously [50] and included examination of intimal hyperplasia, inflammatory cell infiltration, subintimal fibrosis and vascularity with scores ranging from 0 to 3 for each criterion and a range of aggregate scores between 0 and 12.

### Chondrocyte isolation and culture

The clinical features of five patients who were diagnosed according to the criteria of the American College of Rheumatology [51] are summarized in Table 1. Articular cartilage samples were obtained from OA patients who underwent arthroplastic knee surgery. And rat joint cartilage samples were harvest from ACLT + PBS, ACLT + melatonin, ACLT + IL-1 ra and control groups. Primary chondrocytes were isolated as described previously [10]. The human or rat tissues were carefully collected through surgery from the resected bone and cartilages, cut into small pieces, and washed with PBS. The cartilage pieces were digested with 1.5 mg/ml collagenase II in DMEM at 37°C overnight on a shaking platform. The solution was centrifuged at 800 *g* for 8 min and washed thrice with PBS. The pellet was suspended in DMEM containing 10% FBS, 2 mM L-glutamine, 25 mM HEPES, 100 U/ml penicillin, and 100 mg/ml streptomycin at 37°C in a humidified atmosphere of 95% air and 5% CO_2_. The cells which maintained chondrocyte-like phenotype were used in all experiments.

**Table 1:** The clinical characteristics of OA patients

Patient number	Age	Gender	Grade of cartilage degeneration	Duration of disease	Osteophyte formation	Dysfunction of joint
1	77	female	moderate OA	>20 years	+	moderate
2	75	female	moderate OA	∼10 years	+	moderate
3	74	female	severe OA	>20 years	+++	severe
4	66	female	severe OA	∼10 years	+++	severe
5	78	male	severe OA	>20 years	+++	severe

### Cell treatments

For *in vitro* experiments, human chondrocytes were seeded into dishes and cultured in an expansion medium until 70% confluence. The medium was replaced by a serum-free medium to minimize any serum-induced effects on chondrocytes. The chondrocytes were stimulated with or without 10 ng/ml of IL-1β for 30 min. The cells were exposed to various concentrations (0.1, 1, 10, and 100 ng/ml) of melatonin in the presence or absence of IL-1β (10 ng/ml) for different durations (6, 12, 24, and 48 h). Cells with neither IL-1β nor melatonin treatment were used as control. FK866, EX527, anti-TNF-α antibody, anti-IL-1β antibody, celecoxib, or 1400W were directly added to subconfluent cells 30 min prior to IL-1β stimulation and incubated for the indicated times to determine their inhibitory effects on chondrocytes.

### Cell viability assay

MTT assay was performed to determine the effects of melatonin on the viability of human chondrocytes. In brief, chondrocytes were seeded in a 96-well plate at a density of 6 × 10^3^ cells/well. The cells were stimulated with IL-1β (10 ng/ml) for 30 min prior to 24 h of melatonin treatment with various concentrations (0.1, 1, 10, and 100 ng/ml) or 10 ng/ml melatonin treatment for different durations (6, 12, 24, and 48 h). MTT (5 mg/ml, 20 μl) was added to each well and incubated for additional 4 h at 37°C. The supernatant was removed, and the formazan crystals were dissolved in 200 μl DMSO. The optical density (OD) was recorded using a microplate reader (Molecular Devices, Sunnyvale, CA, USA) at 570 nm.

### Gene silencing

Small-interfering RNAs (siRNAs) specific for Sirt1, NAMPT, or NFAT5 or scrambled siRNAs were purchased from Santa Cruz Biotechnology. Human chondrocytes were plated in six-well plates at a density of 5 × 10^5^ cells/well. Until 70% confluence, the chondrocytes were transfected with 100 nM siRNA by using Lipofectamine 2000 reagent (Invitrogen, Carlsbad, CA, USA).

### Quantitative real-time polymerase chain reaction (qPCR)

Total RNA was extracted from human chondrocytes with various treatments by using TRIzol reagent (Invitrogen) according to the manufacturer's protocol. Reverse transcription was performed using SuperScript™ II Reverse Transcriptase (Invitrogen), and cDNAs were amplified and detected using SYBR Premix Ex Taq™ (TaKaRa, Otsu, Shiga, Japan). The 2^−ΔΔCt^ method was used to determine relative gene expression, and the levels of mRNAs were normalized to those of GAPDH. The primer sequences for PCR amplification are listed in Table 2.

**Table 2:** The primer sequences used for qPCR assay

Gene	Primer sequence (5′-3′)	
Sirt1	Forward	TAGAGAACCTTTGCCTCAT
	Reverse	AAAATGTAACGATTTGGTGG
NAMPT	Forward	GCGGCAGAAGCCGAGTTCAAC
	Reverse	CCCATAAAATACTGTTTC CTC
NFAT5	Forward	AGCCATTCAGTCTTTGCT
	Reverse	GATGGTAGCATAGCACAG
COX-2	Forward	TGAGCATCTACGGTTTGCTG
	Reverse	TGCTTGTCTGGAACAACTGC
iNOS	Forward	CGACGGCACCATCAGAGG
	Reverse	AGGATCAGAGGCAGCACATC
MMP-3	Forward	GCGTGGATGCCGCATATGAAGTTA
	Reverse	AAACCTAGGGTGTGGATGCCTCTT
MMP-13	Forward	AAGGACCCTGGAGCACTCATGTTT
	Reverse	TGGCATCAAGGGATAAGGAAGGGT
GAPDH	Forward	TCA CCA TCT TCC AGG AGCGA
	Reverse	CAC AAT GCC GAA GTG GTCGT

### Chromatin immunoprecipitation (ChIP)

Human chondrocytes subjected to the indicated treatments were cross-linked with formaldehyde and harvested for ChIP assay. Chromatin was fragmented by sonication, and the pre-cleared chromatin was immunoprecipitated overnight with antibody against Sirt1 or its corresponding IgG isotype control. The enrichment of specific DNA fragments was analyzed by PCR with primers flanking the NAMPT and NFAT5 promoter regions. The primer sequences are listed in Table 3.

**Table 3:** List of primer sequences of human genes for semi-quantitative ChIP assay

Gene	Primer sequence (5′-3′)	
NFAT5	Forward	AAACGTCTTTCCTCCAACGA
	Reverse	GGGAGCAGGTACAGTGGGTA
NAMPT	Forward	GAGGATCGGAATCCACAAGA
	Reverse	GGACTGAGGAGGACGTGAG

### Western blot analysis

Cells lysates were prepared from human or rat chondrocytes with the indicated treatments by using RIPA lysis buffer (Beyotime Biotechnology, Jiangsu, China). Protein concentration was determined using Bradford assay (Bio-Rad Laboratories, Hercules, CA, USA). An equal amount (25 μg) of protein was separated by electrophoresis on 12% polyacrylamide gels and electrotransferred onto polyvinylidene fluoride membrane (Millipore, Billerica, MA, USA). The membranes were blocked with 5% non-fat milk at room temperature for 1 h and probed with specific primary antibodies, including Sirt1, NAMPT, NFAT5, COX-2, iNOS, MMP-3, MMP-13, Lamin B, and β-actin, at 4°C overnight. The membranes were then incubated with appropriate horseradish peroxidase-conjugated secondary antibodies for 1 h at 37°C. Bands were visualized using enhanced chemiluminescence detection reagents (Pierce, Rockford, IL, USA). Specific protein signals were quantified using Quantity-One software (Bio-Rad) and normalized to the signal intensity of Lamin B or β-actin.

### Sirt1 activity assay

Sirt1 activity of human chondrocytes with the indicated treatments was assessed using fluorometric assay kits (Abcam) according to the manufacturer's instructions. In brief, the cells were lyzed using RIPA lysis buffer and the lysates (2.5 μg) were incubated with 1 μl of Sirt1 substrate at 37°C for 1 h. The samples were then incubated with capture and detection antibodies and allowed to undergo a 10 min colorimetric reaction with tetra-methylbenzidine substrates. Sirt1 activity was assayed using a microplate reader (Molecular Devices) under excitation at 360 nm and emission at 460 nm. Enzymatic activity was expressed in relative fluorescence units.

### NAD+ measurement

NAD^+^ levels in human chondrocytes with the different treatments were measured using a colorimetric NAD/NADH Assay Kit (BioVision Inc., Milpitas, CA, USA) according to the manufacturer's instructions. Chondrocytes were lysed using NAD/NADH extraction buffer. Half of lysate was used to determine the total NAD concentration. The remaining amount was heated to 60°C for 30 min and used to determine NADH concentration. Absorbance was read at 450 nm on a microplate reader (Molecular Devices). NAD^+^ concentration was calculated by subtracting the NADH concentration from the total NAD concentration.

### ELISA assay

After the indicated treatments, the human chondrocyte supernatants or rat SF were collected for ELISA assays. The levels of TNF-α, IL-1β, PGE_2_, MMP-3, and MMP-13 in the supernatant or rat SF were measured using ELISA kits according to the manufacturer's instructions. Absorbance was read at 490 nm on a microplate reader (Molecular Devices).

### NO production assay

The production of NO was determined by using Griess reagent according to manufacturer's protocols. Human chondrocytes were seeded into 96-well plates and subjected to various treatments. Culture supernatants (50 μl) were harvested and incubated with 50 μl of Griess reagent. NO production was determined through nitrite accumulation using sodium nitrite as standard. OD was measured at 570 nm on a microplate reader (Molecular Devices).

### Statistical analysis

Data were expressed as means ± standard deviation (SD). For parametric data, comparison of different groups was performed by one-way analysis of variance, followed by multiple-comparison Tukey post-hoc tests. Non-parametric data were analyzed by Mann-Whitney U test. Statistical analysis was performed using SPSS version 16.0 software (SPSS Inc., Chicago, IL, USA). *P* < 0.05 was considered significant.

## Abbreviations

ChIP: chromatin immunoprecipitation
COX-2: cyclooxygenase-2
DMEM: Dulbecco's modified Eagle medium
DMSO: dimethylsulfoxide
ECM: extracellular matrix
ELISA: enzyme-linked immunosorbent assay
FBS: fetal bovine serum
HEPES: 2-(4-(2-hydroxyethyl)-1-piperazinyl) ethanesulfonic acid
IL-1β: interleukin-1β
iNOS: inducible nitric oxide synthase
MMPs: matrix metalloproteinases
MTT: 3-(4,5-dimethylthiazol-2-y1)-2,5-diphenyltetrazolium bromide
NAD^+^: nicotinamide adenine dinucleotide
NAMPT: nicotinamide phosphoribosyltransferase
NFAT5: nuclear factor of activated T cells 5
NO: nitric oxide
OA: osteoarthritis
OD: optical density
PBS: phosphate-buffered saline
PGE_2_: prostaglandin E2
qPCR: quantitative real-time polymerase chain reaction
SD: standard deviation
Sirt1: sirtuin 1
siRNA: small interfering RNA
TNF-α: tumor necrosis factor-α
